# Hours of Sleep

**Published:** 1892-04

**Authors:** 


### HOURS OF SLEEP.

Man, in common with most of the animal creation, has accepted the plain suggestion of nature that the approach of night should imply a cessation of effort. If he ignores this principle, his work is done against inherited habit, and, so far, with additional fatigue. It follows, too, that he must use artificial light and sustain its combustion at the cost of his own atmosphere. Naturally, therefore, when he does rest, his relief is not proportioned to his weariness. As in many cases, however, sensation is not here the most reliable guide to judicious practice. Established custom affords a far truer indication of the method most compatible with healthy existence. The case of the overworked and the invalid lends but a deceptive color to the argument of the daylight sleeper. In them excessive waste of tissue must be made good, and sleep, always too scanty, is at any time useful for this purpose. For the healthy majority, however, the old custom of early rest and early waking is certain to prove in future—as returns of longevity and common experience alike show that it has proved in the past—most conducive to health and active life.—*London Lancet.*

